# First versus second year respiratory syncytial virus prophylaxis in chronic lung disease (2005–2015)

**DOI:** 10.1007/s00431-017-2849-4

**Published:** 2017-01-20

**Authors:** Daniel Y. Wang, Abby Li, Bosco Paes, Ian Mitchell, Krista L. Lanctôt, CARESS Investigators

**Affiliations:** 1 Medical Outcomes and Research in Economics (MORE®) Research Group, Sunnybrook Health Sciences Centre, University of Toronto, 2075 Bayview Avenue, Room FG-21, Toronto, ON M4N 3M5 Canada; 2 Department of Pediatrics, McMaster University, Hamilton, ON Canada; 3 Department of Pediatrics, University of Calgary, Calgary, AB Canada

**Keywords:** Chronic lung disease, Palivizumab, Two seasons, Comparison, Outcomes

## Abstract

Children aged <2 years with chronic lung disease (CLD) have a 10-fold higher risk for respiratory syncytial virus-positive hospitalization (RSVH) compared to healthy term infants. Based on the updated position statements, we compared respiratory-related illness hospitalization (RIH) and RSVH risks in CLD children who received palivizumab during the first year (FY) versus second year (SY) of life in the Canadian Registry of Palivizumab (CARESS). Demographic data were collected at enrolment and RIH events recorded monthly from 2005 to 2015. Eight hundred forty-seven FY and 450 SY children with CLD were identified. SY children had a lower gestational age (27 versus 29 weeks) and required more days of respiratory support (64 versus 43), oxygen therapy (108 versus 55), and length of stay (118 versus 73) during the neonatal course compared to FY children; all *p* < 0.0005. RIH rates were 12.2 (FY) and 18.2 (SY), and RSVH rates were 2.3 (FY) and 3.9 (SY). Cox regression showed similar hazards for both RIH (hazard ratio 0.9, 95% CI 0.6–1.6, *p* = 0.812) and RSVH (hazard ratio 1.1, 95% CI 0.4–2.9, *p* = 0.920).

*Conclusions*: SY and FY children had similar risks for RIH and RSVH. The findings imply that SY children with CLD are correctly selected for palivizumab based on neonatal illness severity and merit prophylaxis.

**Table:** 

**What is Known:**
*• Children with chronic lung disease have a 10-fold higher risk for RSV-positive hospitalization in comparison to healthy term infants and commonly receive palivizumab prophylaxis as a preventative measure against serious RSV-related lower respiratory tract infections.*
*• The American Academy of Pediatrics [* 2 *] and the Canadian Paediatric Society [* 30 *] have recently modified their recommendations for RSV prophylaxis in children with chronic lung disease, limiting palivizumab to either those <32 weeks gestation or those in the first year of life who are oxygen dependent or require medical therapy for the treatment of their condition.*
**What is New:**
*• Children with chronic lung disease receiving an additional course of palivizumab in their second year of life were determined to be at similar risk for both respiratory illness-related hospitalization and RSV-positive hospitalization as palivizumab-naïve children enrolled in the first year of life in the Canadian Registry for palivizumab (CARESS).*
*• CARESS physicians are correctly identifying high-risk children with chronic lung disease in their second year of life, whom they believe will benefit from an additional year of palivizumab prophylaxis, based on neonatal illness severity.*

## Introduction

Lower respiratory tract infections (LRTIs) are the leading cause of post – neonatal mortality, accounting for over 20% of the estimated 2 million global deaths in 2010 [22, 43]. Human respiratory syncytial virus (RSV) is the primary viral respiratory pathogen associated with these LRTIs, and in Canada, the seasonal outbreaks of RSV illness typically occur between November and April. Clinical symptoms are usually mild and well contained by the immune system, but certain children with high-risk underlying medical conditions may experience severe respiratory illness following RSV infection, which necessitates hospitalization and intensive medical care [4, 34, 43].

Chronic lung disease (CLD) in premature infants is an established risk factor for RSV-related infection with subsequent complications [9, 25]. Children with CLD endure prolonged lengths of hospital stay following RSV-related LRTI with concomitant use of antibiotics, diuretics, bronchodilators, steroids, respiratory support, and ventilation. The risk of RSV-positive hospitalization (RSVH) in the IMpact-RSV study was 12.8% for children with CLD [37]. The mortality rate due to RSV disease in children with CLD is generally low (ranging between 0 and 10%) with a weighted mean rate of 4.1%, but rates as high as 23% have been reported in earlier literature [3, 30, 36, 42].

Palivizumab (Synagis®) is a safe, humanized monoclonal antibody that has been approved for prophylaxis against serious LRTIs caused by RSV infection in high-risk infants [3, 10, 31, 37, 43]. Major indications for palivizumab include CLD, hemodynamically significant congenital heart disease, and prematurity [3, 18]. To date, studies conducted in several countries have demonstrated the efficacy of palivizumab in the reduction of RSVH in children <2 years of age with CLD [9, 11, 14, 24, 26, 33, 37]. The American Academy of Pediatrics (AAP) in 2012 and the Canadian Paediatric Society (CPS) in 2009 had recommended palivizumab for all CLD children <2 years of age for the prevention of RSV-related infection [1, 32]. However, because the incidence of RSVH in children with CLD declines in the second year of life, the cost-effectiveness of palivizumab in children aged >1 year has been debated [5, 35]. As a result, current AAP (2014) and CPS (2015) guidelines limit RSV prophylaxis for CLD patients in their second year of life to only those who are oxygen dependent in the second year [30] or have received at least 28 days of supplemental oxygen after birth and who continue to require medical therapy between 1 and 2 years of age [11].

Based on the recent, more restrictive guidelines for RSV prophylaxis in children with CLD, the primary objective of our study was to compare the risks of respiratory illness-related hospitalizations (RIHs) and RSVH in children with CLD receiving prophylaxis with palivizumab in their first versus second year of life.

## Materials and methods

The Canadian RSV Evaluation Study of Palivizumab (CARESS) is an ongoing prospective, longitudinal, non-randomized, observational, open-cohort study that was launched in 2005. The registry monitors the national usage and adherence patterns of palivizumab in addition to identifying determinants of respiratory illness-related outcomes in children at high risk of RSV infection. Currently, there are a total of 32 collaborating hospitals across eight Canadian provinces, and any child who receives at least one injection of palivizumab at one of these sites is eligible for enrollment. The majority of hospitals function similarly and provide prophylaxis through institution operated RSV clinics. Children are excluded from the study if they are receiving palivizumab within a clinical trial or if their parents or legal guardians are not capable of communicating in English or French. Research ethics board approval was obtained for the study at each participating site and investigational procedures conform to the Declaration of Helsinki guiding principles.

Informed consent is obtained from caregivers of all participating subjects prior to study enrollment. Once enrolled, baseline information regarding demographics, medical history, neonatal course, and palivizumab administration are collected by the treating physician or research nurse at each site. Monthly injections of palivizumab are accompanied by subsequent follow-up telephone interviews to obtain data on palivizumab utilization and adherence, changes in baseline data, adverse events, and any other complications associated with a respiratory infection event encountered after the previous interview. Monthly follow-up interviews are continued until 1 month after the end of the relevant RSV season.

In the event of a hospitalization, relevant details are extracted from patient hospital records by the site’s research nurse following parental or legal guardian approval. Collected data consisted of, but was not limited to, length of stay, diagnoses, RSV confirmation, and requirement of respiratory support, oxygen therapy, or intubation. RSV diagnosis is established by polymerase chain reaction, enzyme or immunofluorescent assay, or viral culture on nasopharyngeal swabs, aspirates, or washes obtained from the patients during their hospital stay. An RIH with a positive RSV test result by any of the detection methods mentioned above is categorized as an RSVH.

### Patient selection and outcome definitions

Chronic lung disease and its severity were defined a priori using criteria established by the National Institute of Child Health and Human Development consensus guidelines [40]. Subjects who presented with CLD as the primary indication for palivizumab prophylaxis were selected from the CARESS population for this study, regardless of any other coexisting medical morbidity such as congenital heart disease or cystic fibrosis. Only children <24 months of age were included in the analyses in order to conform to pediatric advisory guidelines. This cohort was further sub-classified into two groups based on age. Children in the first year (FY) of life were defined as those who were <12 months of age without previous palivizumab prophylaxis. Children in the second year (SY) of life were between 12 and 24 months of age and had received palivizumab during the first year of life. Study subjects were considered adherent to the palivizumab prophylaxis protocol if they received either ≥5 or at least the expected number of palivizumab injections within the recommended time intervals between injections [8]. This definition comprehensively accounts for the full duration of the RSV season as well as the therapeutically effective inter-dose interval derived from pharmacokinetic studies utilizing palivizumab [19, 29]. The recommended time intervals were 16–35 days between the first and second injections and 25–35 days between subsequent injections.

A sub-analysis was conducted to compare the effect of seasonal palivizumab prophylaxis on RIH and RSVH risk in children with CLD. This involved a re-categorization of the CLD population into two new groups based on exposure to the number of RSV seasons. Children who received prophylaxis during their first RSV season were classified as first season (FS) while those who received prophylaxis for two consecutive RSV seasons were defined as second season subjects (SS).

Based on the 2009 CPS guideline, pediatricians provided palivizumab for children <2 years of age with CLD [40], who required ongoing medical therapy (such as oxygen, steroids, or bronchodilators) within the 6 months preceding the RSV season [32]. The majority of CLD children in the FY group qualified for RSV prophylaxis because they were oxygen dependent for the first 28 days of life and/or required respiratory support while those in the SY group required ongoing oxygen or drug therapy for the management of their underlying CLD.

### Statistical analyses

Data were analyzed using IBM SPSS Statistics v20.0 (IBM Corp., Armonk, NY). Baseline demographics, neonatal characteristics, and hospitalization severity markers were compared between the two groups using the Pearson chi-squared test for categorical variables and the Mann-Whitney *U* test for continuous variables. Median values with their corresponding interquartile range (IQR) were reported for continuous variables unless indicated otherwise. An asymptotic *p* value of less than 0.05 was considered to be statistically significant. For samples with an expected count of less than 5, the exact asymptotic *p* values were reported instead.

For descriptive purposes, RIH and RSVH rates were calculated. To determine the RIH rate, the number of children hospitalized for respiratory-related illnesses was divided by the total number of children in the study population. The RSVH rate was calculated by multiplying RIH by the number of RSV-positive children divided by the number of children tested for RSV infection.

Cox proportional hazard regressions using a backward conditional method were performed to evaluate the relative risk of RIH and RSVH between FY and SY children, with the FY ﻿group as the reference category. The time factor was the number of days from enrolment to the patient’s first RIH or RSVH. To account for potential confounders that may affect the time to hospitalization, all demographic and neonatal variables that were found to be significantly different between the two groups were adjusted for as covariates. In cases where multiple potential covariates were highly correlated, only the most significantly predictive variable was included to multicollinearity. Hazard ratio (HR), 95% confidence interval (95% CI), and *p* value are reported for each individual regression analysis. The same set of statistical analyses was applied in the sub-analysis to assess outcomes based on exposure to one or two consecutive RSV seasons after birth.

## Results

Eight hundred forty-seven (65.3%) and 450 (34.7%) of 1297 CLD children received palivizumab prophylaxis during the first and second year, respectively. The median age of children at enrolment was 6.4 months (interquartile range [IQR] 3.7–8.0 months) for the FY group and 16.2 months (IQR 14.0–19.3 months) for the SY group. On average, the children received 4.8 ± 1.2 injections. One thousand fifty-six (81.4%) received at least all of their expected injections, and 956 (73.7%) were adherent to the inter-dose interval protocol. Eight hundred fifteen (62.8%) children were considered fully adherent based on both adherence definitions. Table 1 compares the baseline demographics between FY and SY groups. A higher proportion of SY children attended daycare (11.6 versus 2.8%, *p* < 0.0005) and were of multiple birth status (26.9 versus 21.1%, *p* = 0.019) compared to FY children. Children in the second versus first year of life also had a shorter length of gestation (27.0 [25.1–31.7] versus 29.0 [26.1–37.0] weeks, *p* < 0.0005) and weighed less at birth (880 [698–1453] versus 1152 [790–2671] g, *p* < 0.0005). Additionally, SY infants experienced a significantly more complicated neonatal course as shown in Table 2. They required prolonged respiratory support and oxygen therapy with an extended neonatal hospital stay following birth (all *p* < 0.0005). Furthermore, they sustained greater complications such as necrotizing enterocolitis, and a higher percentage of SY infants also underwent surgery for patent ductus arteriosus (all *p* < 0.05).

**Table 1:** Demographic characteristics of CLD patients in the first and second year of life (*n* = 1297)

	First year (*n* = 847)	Second year (*n* = 450)	*χ* ^2^ or *U*	*p* value*
Male, *n* (%)	483 (57.0)	253 (56.2)	0.08	0.781
Caucasian, *n* (%)	596 (70.4)	306 (68.0)	0.78	0.378
Daycare attendance, *n* (%)^a^	24 (2.8)	52 (11.6)	40.5	<0.0005
Siblings, *n* (%)	519 (61.3)	290 (64.4)	1.26	0.262
Mother smokes, *n* (%)	116 (13.7)	57 (12.7)	0.27	0.604
Mother smoked during pregnancy, *n* (%)	108 (12.8)	46 (10.2)	1.84	0.176
Smokers in the household, *n* (%)	219 (25.9)	112 (24.9)	0.15	0.704
≥2 smokers in the household, *n* (%)	76 (9.0)	35 (7.8)	0.54	0.464
>5 people in the household, *n* (%)	165 (19.5)	100 (22.2)	1.36	0.244
Family history of atopy, *n* (%)	379 (44.9)	205 (45.7)	0.07	0.796
Multiple birth, *n* (%)^a^	179 (21.1)	121 (26.9)	5.48	0.019
Enrolment age, months (median [IQR])	6.4 (3.7–8.0)	16.2 (14.0–19.3)	381,150	<0.0005
Gestational age, weeks (median [IQR])^a^	29.0 (26.1–37.0)	27.0 (25.1–31.7)	147,996	<0.0005
Birth weight, g (median [IQR])^a^	1152 (790–2671)	880 (698–1453)	145,582	<0.0005
Enrolment weight, g (median [IQR])	5640 (4405–6950)	8860 (7752–9960)	341,962	<0.0005

*CLD* chronic lung disease, *IQR* interquartile range

*p value <0.05 is significant

^a^Variables controlled for in Cox proportional hazard regression analyses

**Table 2:** Events incurred by the CLD patients during the neonatal period (*n* = 1297)

	First year (*n* = 847)	Second year (*n* = 450)	*χ* ^2^ or *U*	*p* value*
Length of neonatal stay, days (median [IQR])^a^	73.0 (24.0–110)	118 (81.0–170)	242,996	<0.0005
Respiratory support, *n* (%)^a^	642 (75.8)	378 (84.0)	11.8	0.001
Days on respiratory support (median [IQR])	43.0 (14.0–72.0)	64.0 (37.0–90.0)	151,806	<0.0005
Oxygen therapy, *n* (%)	719 (84.9)	392 (87.1)	1.18	0.277
Days on oxygen therapy (median [IQR])^a^	54.5 (21.0–101)	108 (56.0–202)	174,959	<0.0005
Documented necrotizing enterocolitis, *n* (%)^a^	41 (4.8)	36 (8.0)	5.25	0.022
Surgery for patent ductus arteriosus, *n* (%)^a^	116 (13.7)	110 (24.4)	23.6	<0.0005
Documented sepsis, *n* (%)^a^	226 (26.7)	171 (38.1)	18.0	<0.0005

*CLD* chronic lung disease, *IQR* interquartile range

*p value <0.05 is significant

^a^Variables controlled for in Cox proportional hazard regression analyses

Of the 1297 CLD subjects included in this study, 185 children required a total of 239 hospitalizations for respiratory illnesses after being discharged from the neonatal unit, resulting in a RIH rate of 14.3%. The RIH rate for each group was 12.2 and 18.2% for FY and SY children, respectively (Table 3). Significant differences during the hospital course for RIH were observed between the two groups in terms of the diagnosis of bronchiolitis (FY 61.3% versus SY 40.0%, *p* = 0.001), requirement of respiratory support (FY 34.3% versus SY 22.5%, *p* = 0.048), and requirement for intubation (FY 8.8% versus SY 1.0%, *p* = 0.009). Length of respiratory support was also significantly longer (*p* = 0.049) for FY children compared to SY children. Eighteen and 15 children tested positive for RSV infection in the FY and SY groups, respectively. The RSVH rate was 2.3% for the FY group and 3.9% for the SY group, giving an overall RSVH rate of 2.9% (Table 3). RSVH severity did not differ significantly between the first and second year children. Full details on in-hospital events following RIH or RSVH are depicted in Tables 4 and 5, respectively.

**Table 3:** Number of patients hospitalized for RIH and RSVH (unadjusted rates)

	First year (*n* = 847)	Second year (*n* = 450)	Total (*n* = 1297)
Hospitalized	103	82	185
Tested	94	70	164
RSV-positive	18	15	33
RIH rate (%)	12.2	18.2	14.3
RSVH rate (%)	2.3	3.9	2.9

*RIH* respiratory illness-related hospitalization, *RSV* respiratory syncytial virus, *RSVH* RSV-positive hospitalization

**Table 4:** Events in CLD patients following RIH (*n* = 239)

	First year (*n* = 137)	Second year (*n* = 102)	*χ* ^2^ or *U*	*p* value*
Apnea, *n* (%)	4 (2.9)	2 (2.0)	0.21	>0.999
Bronchiolitis, *n* (%)	84 (61.3)	40 (40.0)	10.5	0.001
Decreased oxygen saturation, *n* (%)	70 (52.2)	53 (53.5)	0.04	0.845
Inability to maintain oral intake, *n* (%)	57 (41.6)	43 (42.6)	0.02	0.881
Pneumonia, *n* (%)	54 (39.4)	45 (44.1)	0.53	0.465
Respiratory arrest, *n* (%)	1 (0.7)	1 (1.0)	0.05	>0.999
Respiratory distress, *n* (%)	98 (71.5)	83 (81.4)	3.08	0.079
Respiratory support required, *n* (%)	47 (34.3)	23 (22.5)	3.90	0.048
Days on respiratory support (median [IQR])^a^	6.0 (2.0–8.0)	5.0 (2.0–9.0)	5608	0.049
Admission to intensive care unit, *n* (%)	29 (21.2)	15 (14.7)	1.63	0.202
Days in intensive care (median [IQR])^a^	6.0 (3.0–12.0)	3.0 (2.0–9.0)	6464	0.143
Intubation required, *n* (%)	12 (8.8)	1 (1.0)	6.88	0.009
Days of intubation (median [IQR])^a^	8.5 (6.5–27.5)	10.0	6446	0.009
Length of hospital stay in days (median [IQR])	5.0 (2.0–9.0)	4.0 (2.0–7.0)	5871	0.117

*CLD* chronic lung disease, *RIH* respiratory illness-related hospitalization, *IQR* interquartile range

*p value <0.05 is significant

^a^Median and IQR are reported only for those who received respiratory support, intensive care, or intubation. All patients who experienced a RIH were included in the Mann-Whitney *U* test to obtain the *U* statistics and corresponding *p* values

**Table 5:** Events in CLD patients following RSVH (*n* = 33)

	First year (*n* = 18)	Second year (*n* = 15)	*χ* ^2^ or *U*	*p* value*
Apnea, *n* (%)	0	1 (6.7)	1.24	0.455
Bronchiolitis, *n* (%)	16 (88.9)	11 (73.3)	1.33	0.375
Decreased oxygen saturation, *n* (%)	8 (47.1)	7 (46.7)	0.00	>0.999
Inability to maintain oral intake, *n* (%)	7 (38.9)	9 (60.0)	1.46	0.227
Pneumonia, *n* (%)	5 (27.8)	6 (40.0)	0.55	0.458
Respiratory arrest, *n* (%)	0	1 (6.7)	1.24	0.455
Respiratory distress, *n* (%)	10 (55.6)	10 (66.7)	0.42	0.515
Respiratory support required, *n* (%)	8 (44.4)	3 (20.0)	2.20	0.138
Days on respiratory support (median [IQR])^a^	9.0 (4.0–28.0)	3.0 (3.0–7.0)	90.0	0.180
Admission to intensive care unit, *n* (%)	4 (22.2)	4 (26.7)	0.09	>0.999
Days in intensive care (median [IQR])^a^	25.5 (5.5–47.5)	4.0 (2.0–7.5)	136	>0.999
Intubation required, *n* (%)	3 (16.7)	0	2.75	0.233
Days of intubation (median [IQR])^a^	44.0 (5.0–51.0)	–	113	0.421
Length of hospital stay in days (median [IQR])	6.5 (4.0–14.0)	5.0 (2.0–8.0)	93.0	0.135

*CLD* chronic lung disease, *RSVH* RSV-positive hospitalization, *IQR* interquartile range

* *p* value <0.05 is significant

^a^Median and IQR are reported only for those who received respiratory support, intensive care, or intubation. All patients who experienced a RSVH were included in the Mann-Whitney *U* test to obtain the *U* statistics and corresponding *p* values

**Fig. 1:**
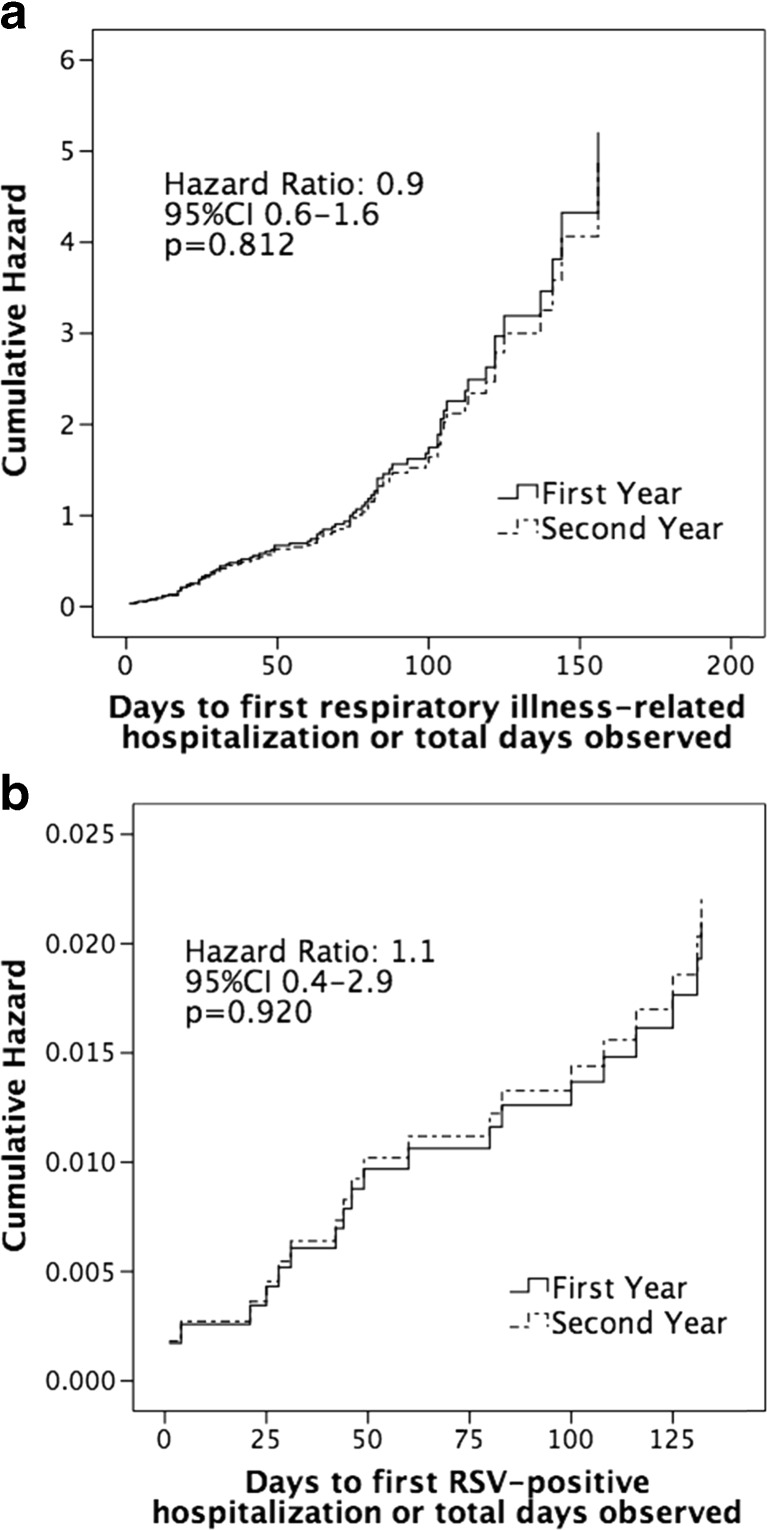
Cox regression for time to first respiratory illness-related hospitalization (**a**) and RSV-specific hospitalization (**b**), comparing children in the first year (*solid line*) versus second year of life (*dotted line*), adjusted for demographics and neonatal course differences indicated with an asterisk in Tables 1 and 2. *RSV* respiratory syncytial virus, *CI* confidence interval

Cox proportional hazard analysis was performed adjusting for daycare attendance, multiple birth status, gestational age, birth weight, medical comorbidities, and neonatal events. Hazard plots are presented in Fig. 1a for RIH and Fig. 1b for RSVH. Children who received palivizumab for the second year displayed a similar risk of both RIH (HR 0.9, 95% CI 0.6–1.6, *p* = 0.812) and RSVH (HR 1.1, 95% CI 0.4–2.9, *p* = 0.920) as those who received palivizumab for only the first year.

The sub-analysis compared a total of 1684 children with CLD; 935 (55.5%) were children who received palivizumab in the first season and 749 (44.5%) received prophylaxis additionally in the second RSV season. Overall, results from this sub-analysis were analogous to those obtained from the primary year of life-based analysis. No effect of seasonality was observed on the times to first RIH (HR 0.9, 95% CI 0.6–1.4, *p* = 0.766) or RSVH (HR 1.3, 95% CI 0.6–2.7, *p* = 0.555).

## Discussion

Children with CLD are particularly vulnerable to serious lower respiratory tract illness following RSV infection and are at heightened risk for hospitalization. In a multicenter Spanish study involving 584 premature (≤32 weeks of gestation) infants, among which 38 (6.5%) were diagnosed with CLD, Carbonell-Estrany et al. found that CLD was a significant independent prognostic variable for the risk of RSVH (odds ratio [OR] 3.1, 95% CI 1.2–7.9, *p* < 0.016) [7]. Liese et al. in a German population-based cohort study confirmed that the RSV-related re-hospitalization rate was greater by 4.0-fold (95% CI 1.4–11.2, *p* = 0.009) in children with CLD compared to those who were not hospitalized for RSV [21]. Similarly, Lee et al. documented that infants with CLD compared to those without CLD were at significantly increased risk for RSVH (OR 2.95; 95% CI 1.44–6.04; *p* = 0.003) [20]. Homaira et al. further confirmed that the incidence of RSVH per 1000 child-years was 81.5 for children with CLD, which was the highest among all cohorts of high-risk children assessed [17]. Likewise, in a large retrospective cohort study, Boyce et al. reported that children with CLD in their first year of life were 10.7 times (95% CI 8.4–13.6) more likely to be hospitalized for RSV infection compared to healthy term infants [5]. A 20-fold (95% CI 11.1–33.7) increase in RSVH risk was also documented for children with CLD during their second year of life relative to low-risk infants of the same chronological age [5]. Furthermore, children with CLD in each age stratum were identified to have the highest RSVH rate among all investigated risk groups, which distinctly signified the susceptibility of children with CLD to severe RSV disease up to 2 years of age compared to other infant populations. Ironically, the evidence from this retrospective study prompted the recent CPS guideline update to limit palivizumab prophylaxis in children with CLD in their second year of life to those still on or weaned off supplemental oxygen in the 3 months prior to the onset of the RSV season [30]. Boyce et al. indeed recorded that the incidence of RSVH in CLD children declined from an estimated 38.8% (95% CI 30.4–49.0%) in those <12 months of age to 7.3% (95% CI 4.2–11.9%) at 12 to 24 months, with a further decrease to 1.3% (95% CI 0.2–4.6%) in the third year of life [5, 30]. It is important to acknowledge that the data from two decades ago may be outdated and inconsistent with the current epidemiology of RSV infection. However, more recent data continue to demonstrate that CLD remains a dominant risk factor governing RSVH [17, 20, 21, 28]. Moreover, the estimated 7.3% RSVH rate for children with CLD in the second year of life [5] is not low enough to justify a guideline change [11] since the average RSVH rate in CARESS for prophylaxed CLD subjects in the second year of life still remained 3.9% in this study and 8.9% in the Korean study conducted by Han et al. [16] in 2007. The Cox analysis from our study further emphasizes that children with CLD over the age of 12 months remain at risk for both RIH and RSVH.

New guideline recommendations denigrate the fact that palivizumab prophylaxis in children with CLD is beneficial up to 2 years of age. In the IMpact-RSV study, palivizumab prophylaxis in children with CLD <24 months of age was shown to reduce the incidence of RSVH by a factor of 39% compared to subjects who received placebo (*p* = 0.038) [37]. Pedraz et al. demonstrated in a Spanish observational cohort study that children aged <2 years with CLD were approximately four times less likely to experience RSVH (5.5 versus 19.7%, *p* < 0.007) if they received palivizumab prophylaxis [26]. Similarly, in a Korean retrospective study involving 128 children with CLD <2 years of age, the RSVH rate was determined to be significantly lower (*p* < 0.001) in patients who received palivizumab (4.0%) than those who did not (22.6%) [9]. In our study, in-hospital events such as length of hospital stay and admission to the intensive care unit following re-hospitalization for respiratory-related and RSV-positive illnesses were similar between the two CLD groups regardless of age and previous palivizumab exposure. While the prophylactic effect was not expected across a duration of 2 years due to the short mean half-life of palivizumab (20 days) and passive protection offered [44], our results imply that the risk of RIH and RSVH in second year CLD patients cannot be neglected. Interestingly, children who received palivizumab only in the first year of life required significantly more respiratory support and a longer duration of intubation for RIH events, which may reflect an increased susceptibility to overall severe LRTI in association with CLD. In contrast, children who received prophylaxis for 2 years had a milder, but statistically insignificant, course of RSV-related illness with lower rates and duration of respiratory support and need for ventilation.

It is equally important to recognize that healthy preterm infants have significant lung immaturity and compromised immune function, which pose a risk for obstructive lung function which persists until 2 years of age and even in adolescents up to 17 years [13, 23, 39]. Moreover, preterm infants with and without CLD, who experience symptomatic RSV LRTI at <2 years of age, have higher airway resistance at 36 weeks post menstrual age, 1 year of life, and at 8–10 years [6, 12, 15]. This implies that infants with CLD, irrespective of gestational age [35] or disease severity [11, 40], are at increased risk for serious RSV disease and merit prophylaxis for two RSV seasons (FY and SY) based on either oxygen need or medical therapy dependency for the management of their CLD status. The use of oxygen alone as a criterion for prophylaxis in the second year minimizes the pathophysiology of CLD and is a relatively weak marker when used solely to discriminate CLD disease severity [27]. This study confirms that SY infants are correctly selected for RSV prophylaxis since they were of lower gestational age than FY infants with lower birth weight (both *p* < 0.0005). In addition, they required significantly longer durations of respiratory support and oxygen therapy, and their neonatal course (days) was more prolonged with complications involving necrotizing enterocolitis, sepsis, and surgery for patent ductus arteriosus.

There are currently no studies that directly compare the cost-effectiveness of palivizumab in CLD children in the FY versus SY. In the UK, Wang et al. reported that the incremental cost-effectiveness ratio (ICER)/quality-adjusted life-year (QALY) for palivizumab prophylaxis, compared with no prophylaxis in CLD children, was £66,900 [41]. Cost-effectiveness varied between £51,000 and £85,000/QALY for mortality rates of 0.05 and 0.03, respectively [42]. Under the current willingness-to-pay threshold of £30,000/QALY, palivizumab was deemed not cost-effective [41, 42]. More recently, Bentley et al. [3] estimated a £19,168/QALY for the use of palivizumab compared with no palivizumab in children with CLD in the UK, which was deemed to be an economically acceptable use of National Health Service resources. Thomas further confirmed in a cost-benefit, sensitivity analysis using the English Hospital Episode Statistics (HES) data set that palivizumab represents good use of health resources for children with CLD in the UK [38]. In essence, the value of prophylaxis in CLD children varies depending on the type of analysis conducted and the population studied. Further studies are required to elucidate the true value of palivizumab in children with CLD.

This study has a number of limitations that should be addressed. First, as with all observational registry studies, the magnitude of the effect of prophylaxis in first versus second year CLD populations cannot be estimated due to the absence of a comparative control arm. Additionally, children with CLD recruited for the SY group were clearly predisposed with a greater illness severity during their neonatal course as depicted in Table 2 compared to palivizumab-naïve FY children, which could have overestimated both the RIH and RSVH risk in SY infants. However, this may simply signal, instead, that CARESS physicians meticulously identified high-risk children with CLD in the second year of life, whom they believe will benefit from an additional year of palivizumab prophylaxis because of continued oxygen dependency and/or medical therapy (diuretics, bronchodilators, steroids) for the management of CLD. On the contrary, RSVH may have been underestimated due to the fact that not every child was tested for RSV following their hospitalization for respiratory-related illnesses. Children in the second year of life may also have acquired some immunity through repetitive exposure to RSV infection. However, the resulting immunoprotective effect was likely subdued since the hazard ratio for RSVH in the second year was statistically indifferent to those children who only received prophylaxis in the first year of life. Furthermore, physicians could have a positive bias toward admitting children with a more protracted neonatal course which may influence the RSVH rate in the second year of life. However, the majority of hospitals use specific criteria to determine need for admission which perhaps dampened the magnitude of this effect. Lastly, despite the large sample size, the low number of hospitalized children in each group may have underpowered the comparison of RIH and RSVH severity markers.

In summary, children with CLD recruited over the 10 years of the CARESS study were compared for the risk of RIH and RSVH. Children aged >1 year who received an additional course of palivizumab for CLD had a similar hazard of both RIH and RSVH as those who received palivizumab only in the first year of life. The findings imply that SY children with CLD are correctly selected for RSV prophylaxis by healthcare providers based on neonatal illness severity and deserve consideration for prophylaxis beyond the stipulated criteria suggested by some national advisory guidelines for palivizumab. Future cost-utility and cost-effectiveness studies are required to examine the economic burden posed by children with CLD in the second year of life compared to those less than 1 year of age.

## Abbreviations

CARESS: Canadian RSV Evaluation Study of Palivizumab
CLD: Chronic lung disease
FY: First year
ICER: Incremental cost-effectiveness ratio
LRTI: Lower respiratory tract infection
QALY: Quality-adjusted life-year
RIH: Respiratory illness-related hospitalization
RSV: Respiratory syncytial virus
RSVH: RSV-positive hospitalization
SY: Second year

## References

[1] Pickering LK, Baker CJ, Kimberlin DW, Long SS, American Academy of Pediatrics (2012). Respiratory syncytial virus. Red book: 2012 report of the Committee of Infectious Diseases.

[2] American Academy of Pediatrics Committee on Infectious Diseases (2014) Updated Guidance for Palivizumab Prophylaxis Among Infants and Young Children at Increased Risk of Hospitalization for Respiratory Syncytial Virus Infection. Pediatrics 134: 415-420 10.1542/peds.2014-1665 25070315

[3] Bentley A, Filipovic I, Gooch K, Büsch K (2013). A cost-effectiveness analysis of respiratory syncytial virus (RSV) prophylaxis in infants in the United Kingdom. Health Econ Rev.

[4] Borchers AT, Chang C, Gershwin ME, Gershwin LJ (2013). Respiratory syncytial virus—a comprehensive review. Clin Rev Allergy Immunol.

[5] Boyce TG, Mellen BG, Mitchel EF, Wright PF, Griffin MR (2000). Rates of hospitalization for respiratory syncytial virus infection among children in Medicaid. J Pediatr.

[6] Broughton S, Bhat R, Roberts A, Zuckerman M, Rafferty G, Greenough A (2006). Diminished lung function, RSV infection, and respiratory morbidity in prematurely born infants. Arch Dis Child.

[7] Carbonell-Estrany X, Quero J, Bustos G, Cotero A, Doménech E, Figueras-Aloy J, Fraga JM, García LG, García-Alix A, Del Río MG (2000). Rehospitalization because of respiratory syncytial virus infection in premature infants younger than 33 weeks of gestation: a prospective study. IRIS Study Group. Pediatr Infect Dis J.

[8] Chan P, Li A, Paes B, Abraha H, Mitchell I, Lanctôt KL, CARESS investigators (2015). Adherence to palivizumab for respiratory syncytial virus prevention in the Canadian registry of palivizumab. Pediatr Infect Dis J.

[9] Chang SG, Park MS, Yu JE (2010). Outcomes of palivizumab prophylaxis for respiratory syncytial virus infection in preterm children with bronchopulmonary dysplasia at a single hospital in Korea from 2005 to 2009. J Korean Med Sci.

[10] Chen JJ, Chan P, Paes B, Mitchell I, Li A, Lanctôt KL, CARESS investigators (2015). Serious adverse events in the Canadian registry of children receiving palivizumab (CARESS) for respiratory syncytial virus prevention. PLoS One.

[11] Committee On Infectious Diseases, Bronchiolitis Guidelines Committee (2014). Updated guidance for palivizumab prophylaxis among infants and young children at increased risk of hospitalization for respiratory syncytial virus infection. Pediatrics.

[12] Drysdale SB, Lo J, Prendergast M, Alcazar M, Wilson T, Zuckerman M, Smith M, Broughton S, Rafferty GF, Peacock JL (2014). Lung function of preterm infants before and after viral infections. Eur J Pediatr.

[13] Friedrich L, Pitrez PMC, Stein RT, Goldani M, Tepper R, Jones MH (2007). Growth rate of lung function in healthy preterm infants. Am J Respir Crit Care Med.

[14] Frogel M, Nerwen C, Cohen A, VaVeldhuisen P, Harrington M, Boron M (2008). Prevention of hospitalization due to respiratory syncytial virus: results from the Palivizumab Outcomes Registry. J Perinatol.

[15] Greenough A, Alexander J, Boit P, Boorman J, Burgess S, Burke A, Chetcuti PA, Cliff I, Lenney W, Lytle T (2009). School age outcome of hospitalisation with respiratory syncytial virus infection of prematurely born infants. Thorax.

[16] Han YM, Seo HJ, Choi SH, Jung YJ, Ahn SY, Yoo HS, Sung SI, Shim JW, Lee YK, Ko SY (2015). Effect of prophylactic palivizumab on admission due to respiratory syncytial virus infection in former very low birth weight infants with bronchopulmonary dysplasia. J Korean Med Sci.

[17] Homaira N, Oei JL, Mallitt KA, Abdel-Latif ME, Hilder L, Bajuk B, Lui K, Ferson M, Nurkic A, Chambers GM (2016). High burden of RSV hospitalization in very young children: a data linkage study. Epidemiol Infect.

[18] Homaira N, Rawlinson W, Snelling TL, Jaffe A (2014). Effectiveness of palivizumab in preventing RSV hospitalization in high risk children: a real-world perspective. Int J Pediatr.

[19] La Via WV, Notario GF, Yu XQ, Sharma S, Noertersheuser PA, Robbie GJ (2013). Three monthly doses of palivizumab are not adequate for 5-month protection: a population pharmacokinetic analysis. Pulm Pharmacol Ther.

[20] Lee JH, Kim CS, Chang YS, Choi J, Committee on Data Collection and Statistical Analysis of the Korean Society of Neonatology (2015). Respiratory syncytial virus related readmission in preterm infants less than 34 weeks’ gestation following discharge from a neonatal intensive care unit in Korea. J Korean Med Sci.

[21] Liese JG, Grill E, Fischer B, Roeckl-Wiedmann I, Carr D, Belohradsky BH, The Munich RSV Study Group (2003). Incidence and risk factors of respiratory syncytial virus-related hospitalizations in premature infants in Germany. Eur J Pediatr.

[22] Lozano R, Naghavi M, Foreman K, Lim S, Shibuya K, Aboyans V, Abraham J, Adair T, Aggarwal R, Ahn SY (2012). Global and regional mortality from 235 causes of death for 20 age groups in 1990 and 2010: a systematic analysis for the Global Burden of Disease Study 2010. Lancet.

[23] McEvoy C, Venigalla S, Schilling D, Clay N, Spitale P, Nguyen T (2013). Respiratory function in healthy late preterm infants delivered at 33–36 weeks of gestation. J Pediatr.

[24] Paes B, Mitchell I, Li A, Harimoto T, Lanctôt KL (2013). Respiratory-related hospitalizations following prophylaxis in the Canadian registry for palivizumab (2005-2012) compared to other international registries. Clin Dev Immunol.

[25] Paes B, Mitchell I, Li A, Lanctôt KL (2012). Respiratory hospitalizations and respiratory syncytial virus prophylaxis in special populations. Eur J Pediatr.

[26] Pedraz C, Carbonell-Estrany X, Figueras-Aloy J, Quero J, IRIS Study Group (2003). Effect of palivizumab prophylaxis in decreasing respiratory syncytial virus hospitalizations in premature infants. Pediatr Infect Dis J.

[27] Poindexter BB, Feng R, Schmidt B, Aschner JL, Ballard RA, Hamvas A, Reynolds AM, Shaw PA, Jobe AH, Prematurity and Respiratory Outcomes Program (2015). Comparisons and limitations of current definitions of bronchopulmonary dysplasia for the prematurity and respiratory outcomes program. Ann Am Thorac Soc.

[28] Ricart S, Marcos MA, Sarda M, Anton A, Muñoz-Almagro C, Pumarola T, Pons M, Garcia-Garcia JJ (2013). Clinical risk factors are more relevant than respiratory viruses in predicting bronchiolitis severity. Pediatr Pulmonol.

[29] Robbie GJ, Zhao L, Mondick J, Losonsky G, Roskos LK (2012). Population pharmacokinetics of palivizumab, a humanized anti-respiratory syncytial virus monoclonal antibody, in adults and children. Antimicrob Agents Chemother.

[30] Robinson JL, Le SN, Canadian Paediatric Society, Infectious Diseases and Immunization Committee (2015). Preventing hospitalizations for respiratory syncytial virus infection. Paediatr Child Health.

[31] Rogovik AL, Carleton B, Solimano A, Goldman R (2010). Palivizumab for the prevention of respiratory syncytial virus infection. Can Fam Physician.

[32] Samson L, Canadian Paediatric Society, Infectious Diseases and Immunization Committee (2009). Prevention of respiratory syncytial virus infection. Paediatr Child Health.

[33] Simoes EAF, Groothuis JR (2002). Respiratory syncytial virus prophylaxis—the story so far. Respir Med.

[34] Sommer C, Resch B, Simões EAF (2011). Risk factors for severe respiratory syncytial virus lower respiratory tract infection. Open Microbiol J.

[35] Stevens TP, Sinkin RA, Hall CB, Maniscalco WM, McConnochie KM (2000). Respiratory syncytial virus and premature infants born at 32 weeks’ gestation or earlier: hospitalization and economic implications of prophylaxis. Arch Pediatr Adolesc Med.

[36] Szabo SM, Gooch KL, Bibby MM, Vo PG, Mitchell I, Bradt P, Levy AR (2013). The risk of mortality among young children hospitalized for severe respiratory syncytial virus infection. Paediatr Respir Rev.

[37] The IMpact-RSV Study Group (1998). Palivizumab, a humanized respiratory syncytial virus monoclonal antibody, reduces hospitalization from respiratory syncytial virus infection in high-risk infants. Pediatrics.

[38] Thomas G (2015). A cost-benefit analysis of the immunisation of children against respiratory syncytial virus (RSV) using the English Hospital Episode Statistics (HES) data set. Eur J Health Econ.

[39] Thunqvist P, Gustafsson PM, Schultz ES, Bellander T, Berggren-Bronström E, Norman M, Wickman M, Melén E, Hallberg J (2016). Lung function at 8 and 16 years after moderate-to-late preterm birth: a prospective cohort study. Pediatrics.

[40] Walsh MC, Szefler S, Davis J, Allen M, Marter LV, Abman S, Blackmon L, Jobe A (2006). Summary proceedings from the bronchopulmonary dysplasia group. Pediatrics.

[41] Wang D, Bayliss S, Meads C (2011) Palivizumab for immunoprophylaxis of respiratory syncytial virus (RSV) bronchiolitis in high-risk infants and young children: a systematic review and additional economic modelling of subgroup analyses. Health Technol Assess 15(5) 10.3310/hta15050 PMC4781126 21281564

[42] Wang D, Cummins C, Bayliss S, Sandercock J, Burls A (2008) Immunoprophylaxis against respiratory syncytial virus (RSV) with palivizumab in children: a systematic review and economic evaluation. Health Technol Assess 12(36) 10.3310/hta12360 19049692

[43] Wegzyn C, Toh LK, Notario G, Biguenet S, Unnebrink K, Park C, Makari D, Norton M (2014). Safety and effectiveness of palivizumab in children at high risk of serious disease due to respiratory syncytial virus infection: a systematic review. Infect Dis Ther.

[44] Zaaijer HL, Vandenbroucke-Grauls CM, Franssen EJ (2002). Optimum dosage regimen of palivizumab?. Ther Drug Monit.

